# Treatment of severe symptomatic hyponatremia

**DOI:** 10.14814/phy2.14265

**Published:** 2019-11-06

**Authors:** Srijan Tandukar, Helbert Rondon‐Berrios

**Affiliations:** ^1^ Division of Transplant Nephrology Thomas E. Starzl Transplant Institute University of Pittsburgh Medical Center Pittsburgh Pennsylvania; ^2^ Renal‐Electrolyte Division University of Pittsburgh Medical Center Pittsburgh Pennsylvania

**Keywords:** Hyponatremia, Antidiuretic hormone, Water diuresis

## Abstract

Hyponatremia is the most common electrolyte abnormality seen in the hospital. Severe symptomatic hyponatremia is associated with grave consequences including cerebral edema, brain herniation, seizures, obtundation, coma, and respiratory arrest. However, rapid correction of chronic severe hyponatremia may lead to osmotic demyelination syndrome (ODS) and even death. Given the serious consequences of severe hyponatremia or its inadvertent overcorrection, it is of paramount importance for the clinician to be aware of the various scenarios in which hyponatremic patients can present and tailor the management strategies accordingly. We present here a case of severe hyponatremia of unknown duration with the presenting plasma sodium level of 95 mmol/L and use it to illustrate the various treatment strategies – proactive, reactive, or rescue therapy – along with the physiological basis to support these approaches.

## Introduction

Hyponatremia occurs when there is an excess of total body water in relation with total exchangeable body sodium and potassium (Edelman et al., 1958). A patient is considered to be hyponatremic when the plasma sodium concentration (PNa) falls below 135 mmol/L. Severe hyponatremia is often defined as PNa level under 120 mmol/L and may lead to seizures, obtundation, coma, and respiratory arrest (Ayus et al., 1985; Sterns et al., 1994; Halawa et al., 2011; Spasovski et al., 2014). When this occurs within 48 h, it is called acute hyponatremia and delay in management may lead to cerebral edema and brain herniation (Spasovski et al., 2014). On the other hand, overly rapid correction of chronic severe hyponatremia, defined as hyponatremia occurring over more than 48 h can lead to catastrophic complications such as osmotic demyelination syndrome (ODS) and even death (Ayus et al., 1985; Sterns et al., 1994). When the onset of hyponatremia is uncertain, it should be managed as chronic hyponatremia as it would be reasonable to err on the side of caution during the treatment of such cases. We present here a case of severe symptomatic hyponatremia with a PNa of 95 mmol/L at the time of presentation to an outside hospital, which was overcorrected above the safe limit requiring treatment for overcorrection when she came to our hospital.

## Case History

A 58‐year‐old Caucasian female with past medical history of depression, anxiety, hypertension, asthma, and chronic alcohol use with history of alcohol withdrawal seizures was initially brought to an outside hospital after she was found to be unconscious by her husband at around 1 AM. Her husband was able to confirm that she had been sober for 7 years but she had relapsed around 9 months back, when she started consuming different kinds of liquor on a daily basis including vodka and beer, although he could not specify the exact amount. She had nausea and two episodes of vomiting the previous day. When the paramedics arrived in her house, she had a witnessed tonic‐clonic seizure which lasted around 2 min and resolved after she received 2.5 mg of intravenous midazolam. She had a second episode of seizure en route to the hospital in the ambulance which was controlled with 2 mg of intravenous lorazepam. Her vitals on presentation were as follows: blood pressure of 112/59, heart rate of 71/min, temperature of 100°F, respiratory rate of 17/min, and oxygen saturation of 100% on room air. She weighed 65.3 kg with a BMI of 23 kg/m^2^. On examination, she was obtunded and not following commands but able to maintain her airway. Her pupils were equal, round, and reactive to light. Her cardiorespiratory and abdominal exam were normal and she did not have any edema. She was withdrawing to pain on all four extremities but had a left‐sided gaze preference, which was a new finding according to her husband.

The patient was put on the Clinical Institute Withdrawal Assessment for Alcohol (CIWA) protocol with intravenous infusion of thiamine, multivitamin, and folic acid solution. She underwent a CT scan of her head which showed symmetric hypodensities of bilateral parietal lobes suggestive of old infarcts and no acute intracranial abnormalities. She also had a CT angiogram which showed no large cerebral vessel occlusions. She was found to have a PNa of 95 mmol/L. She was given 3% hypertonic saline bolus of 100 mL along with 2 mcg of desmopressin that was followed by a brisk diuresis of 2475‐mL urine within the first hour. She was given one additional dose of desmopressin 2 mcg after 8 h. When PNa increased to 103 mmol/L from admission level in around 12 h (PNa change of 8 mmol/L), she was given 200‐mL bolus of intravenous 5 percent dextrose in water (D5W). However, the PNa continued to increase to 106 mmol/L. At this point, the patient was transferred to our center.

**Figure 1:**
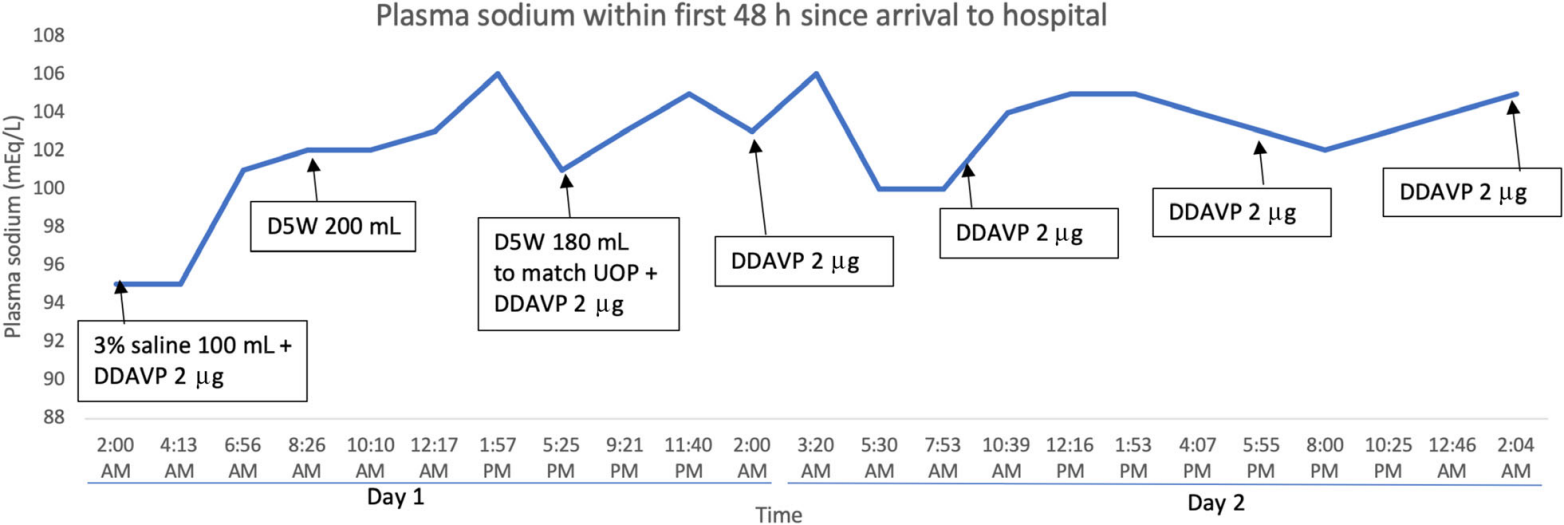
Plasma sodium trends within first 48 hours since arrival to the hospital.

**Figure 2:**
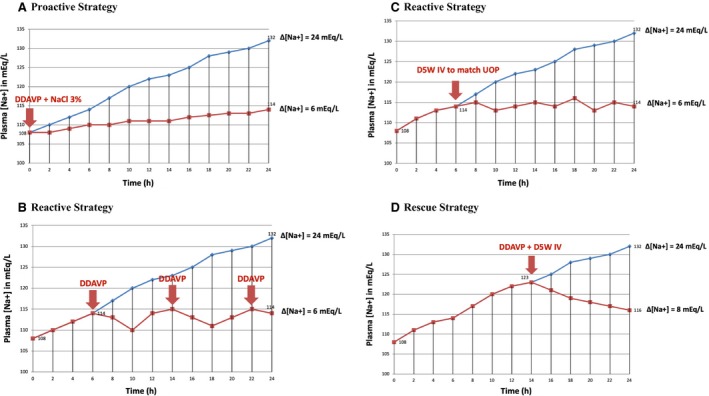
Strategies for management of severe hyponatremia include Proactive Strategy (Panel A) using DDAVP and 3% hypertonic saline; Reactive Strategy (Panel B) using DDAVP, and (Panel C) using D5W to match urine output (UOP); and Rescue Strategy (Panel D) using DDAVP and D5W.

The patient's exam was unchanged on presentation to our hospital. A stat PNa was done and found to be 101 mmol/L. She had a urine output (UOP) of 180 mL within an hour, so 180 mL of D5W was given over the next hour to match UOP to keep the correction of hyponatremia at 6 mmol/L. Desmopressin was continued at the rate of 2 mcg every 8 h. The PNa correction at 24, 48, and 72 h marks was 8, 10, and 16 mmol/L, respectively (Table 1 and Fig. 2). She was discharged after a 12‐day hospital stay after her PNa level slowly corrected to 136 mmol/L at the time of discharge (Fig. 1). Her neurological status was back to baseline at the time of her discharge. Unfortunately, she was lost to follow‐up.

**Table 1:** Key Concepts.

**Hyponatremic patients at high risk for brain herniation** Delayed absorption of ingested water ▪ Use of psychotropic agents such as ecstasy ▪ Psychotic patients with extreme polydipsia ▪ Hypotonic fluid consumption in competitive runners Women and children with acute postoperative hyponatremia Patients with preexisting intracranial pathology High volumes of isotonic fluids postoperatively secondary to syndrome of inappropriate antidiuresis
**Hyponatremic patients at high risk for Osmotic Demyelination Syndrome** PNa < 105 mmol/L Hypokalemia Malnutrition Alcohol use disorder Liver disease
**Hyponatremic patients at high risk for overly rapid correction of plasma sodium (due to water diuresis)** Treatment of low dietary solute intake Treatment of hypovolemia Treatment of cortisol deficiency Resolution of transient SIADH Medications: ▪ Discontinuation of thiazides ▪ Initiation of vasopressin antagonists (vaptans)
**Goals and Limits of Correction of Hyponatremia**
***Goals of correction of hyponatremia*** Change in PNa of 4 to 6 mmol/L in any 24‐hour period
***Limits of correction of hyponatremia*** Change in PNa of < 10‐12 mmol/L in first 24 h and < 18 mmol/L in first 48 h, or Change in PNa < 8 mmol/L in any 24‐hour period

SIADH, Syndrome of Inappropriate Antidiuretic Hormone; SSRI, Selective Serotonin Reuptake Inhibitors; DDAVP, 1‐deamino‐8‐D‐arginine vasopressin; ADH, Antidiuretic Hormone.

## Discussion

Hyponatremia occurs in up to 30% of the hospitalized patients (Spasovski et al., 2014). Despite the high prevalence and grave consequences of this common electrolyte abnormality, there is a paucity of properly conducted prospective randomized controlled trials to guide its treatment (Berl, 2007; Spasovski et al., 2014). The manifestations of hyponatremia may range from headache, fatigue, lethargy, nausea, vomiting, confusion, and disorientation in mild to moderate hyponatremia to the acute onset of seizures, coma, and respiratory arrest in severe hyponatremia (Sterns et al., 1994; Renneboog et al., 2006; Halawa et al., 2011; Spasovski et al., 2014).

## Approach to a Patient with Severe Symptomatic Hyponatremia

All patients with severe symptomatic hyponatremia should be treated in the hospital setting with close monitoring as they are at high risk of developing complications (Ayus et al., 1985; Sterns et al., 1994; Halawa et al., 2011). The severe symptoms of hyponatremia are more common in the acute setting when the brain has not had enough time to adapt to the changes in extracellular tonicity (Sterns et al., 1994; Halawa et al., 2011; Spasovski et al., 2014).

In a retrospective cross‐sectional study done to assess the risk of seizures in hyponatremic patients who did not have a history of epilepsy, investigators found the highest risk of occurrence of seizures at PNa levels below 110 mmol/L with an odds ratio of 18.06 (95% CI: 1.96–166.86) as compared to PNa level of 120–124 mmol/L (Halawa et al., 2011). Another study found that the incidence of seizures was much higher in patients with acute hyponatremia as compared to those with chronic hyponatremia for similar level of severe hyponatremia with PNa less than 110 mmol/L (30% vs 7%) (Sterns, 1987). However, patients with chronic severe hyponatremia who have additional risk factors such as a history of alcohol use disorder and seizures, as in our patient, may be at higher risk of developing these neurologic symptoms than patients with similar PNa level without these risk factors.

The successful management of hyponatremia encompasses: (1) the prevention of further declines in PNa, (2) prevention of brain herniation, (3) relief of symptoms of hyponatremia, and (4) avoidance of overly rapid correction and ODS. It is important to recognize the patients at high risk of further declines in PNa at the outset so that appropriate measures can be taken to limit further worsening of hyponatremia. These patients include: hyponatremia occurring from self‐induced water intoxication who may have a delayed absorption of ingested water, those occurring from use of psychotropic agents such as ecstasy, psychotic patients with extreme polydipsia, and hypotonic fluid consumption in competitive runners (Cheng et al., 1990; Tanneau et al., 1994; Rosner and Kirven, 2007). Such patients are also at high risk of cerebral edema and brain herniation, along with other high‐risk group of patients including women and children with acute postoperative hyponatremia and patients with an intracranial pathology (Ayus et al., 1992; Arieff et al., 1992). Patients who get high volumes of isotonic fluids in the postoperative period may also develop further declines in PNa consequent of the iatrogenic induction of syndrome of inappropriate antidiuresis leading to the excretion of a concentrated urine by a process termed “desalination” (Steele et al., 1997) (Table 1).

Seizures in patients with severe hyponatremia should be managed urgently per routine seizure protocol with antiepileptic medications delivered parenterally. Hypoxic episodes, such as that occurring secondary to a seizure episode, are established risk factors for osmotic demyelination and neurological damage secondary to worsening cerebral edema (Ayus et al., 1987). It is interesting to note that our patient did not suffer from any permanent neurologic sequelae despite having these hypoxic events (two seizure episodes) and other risk factors for ODS. Re‐lowering of plasma sodium to recommended safe limit was perhaps helpful in preventing this complication although we are unable to prove this definitively. Other investigators have also reported therapeutic re‐lowering of PNa after overly rapid correction without any untoward effects on the patient's neurological status and prevention of ODS although these cases had a PNa slightly higher than in our patient (Soupart et al., (1999); Ochiai and Uenishi, 2018). Endotracheal intubation may be required for airway protection. Further management should then focus on careful review of the possible causes of hyponatremia and a step‐wise approach to correction of hyponatremia with close monitoring of PNa levels to avoid overly rapid correction.

## Goals and Limits of Plasma Sodium Correction

This topic has remained a subject of controversy with a general trend toward being more conservative in one's approach to correcting severe symptomatic hyponatremia over the decades. A limit of <10–12 mmol/L within 24 h and <18 mmol/L within 48 h has been recommended by both the US and European guidelines published in 2013 and 2014, respectively (Verbalis et al., 2013; Spasovski et al., 2014). Some investigators have proposed an even more conservative limit of 6 to 8 mmol/L per day (Adrogue and Madias, 2014). In one case series by Ayus et al. (2015), a correction up to 12 to 14 mmol/L in the first 24 to 48 h was found to be safe without evidence of ODS. However, Koul et al. have reported an incidence of ODS even when the rate of correction of PNa was kept ≤ 8 mmol/L per day (Koul et al., 2013). For patients at high risk of ODS, a limit up to 8 mmol/L is advocated by the US guidelines (Verbalis et al., 2013).

There is some variation in the goals of initial therapy recommended by different experts as well, ranging from 4 to 8 mmol/L per day. Some experts have suggested correcting plasma sodium by 4 to 6 mmol/L quickly within 6 hours or less to prevent the occurrence of brain herniation and recurrence of seizure episodes and then keeping it at a constant level in a 24‐h period (Sterns et al., 2010; Sterns, 2018) (Table 1).

The patients at the highest risk of ODS include those with PNa concentration ≤105 mmol/L and those with hypokalemia, malnutrition, alcohol use disorder, and cirrhosis (Verbalis et al., 2007; Berl and Rastegar, 2010; Sterns et al., 2010) (Table 1).

## Strategies to Prevent and Treat Overcorrection of Hyponatremia

The response of an individual patient to the treatment of hyponatremia is variable due to the wide variety of causes leading up to hyponatremia and the possible emergence of water diuresis during treatment which will raise the PNa level more rapidly than conventional formulae may predict (Mohmand et al., 2007; Sterns, 2016). As such, close monitoring of the PNa, initially every 1–2 h, is the cornerstone of successful management of severe symptomatic hyponatremia and prevention of ODS. Such monitoring will also allow selection of one of the following strategies of management in a timely manner or to switch from one strategy to another based on the response to treatment (Fig. 2 and Table 2). We have found the following approach to managing severe hyponatremia to be optimal in our institution's experience.

**Table 2:** Strategies to treat severe hyponatremia.

Strategy	Why?	When?	How?
Proactive	Prevent overcorrection of hyponatremia	Outset of plasma [Na+] correction in patients with: Plasma [Na+]<120 mEq/L High risk for overcorrection High risk for ODS	DDAVP 2–4 mcg IV Q6–8h and 3% NaCl bolus 100 mL over 10 min ×3 as needed (severe symptoms) or 0.5 to 2 mL/kg per hour (moderate symptoms)
Reactive	Prevent overcorrection of hyponatremia	Plasma [Na+] is correcting too fast: Achieved goal of 4–6 mEq/L quickly UOP>100 mL/h	DDAVP 2‐4 mcg IV Q6‐8h or D5W to match UOP cc per cc
Rescue	Treat overcorrection of hyponatremia	Plasma [Na+] overcorrection already occurred	DDAVP 2–4 mcg IV Q6–8h and D5W IV 3 mL/kg per h

### Proactive strategy

This strategy of management is optimal for patients with a PNa < 120 mEq and particularly for patients at risk of overly rapid correction or ODS (Verbalis et al., 2007; Berl and Rastegar, 2010; Sterns et al., 2010) (Table 1 and 2). In such patients, the onset of water diuresis with the treatment of hyponatremia can quickly raise the PNa level leading to the irreversible consequences of ODS. The proactive strategy advocates the use of 3% hypertonic saline intravenously to achieve a slow and sustained rise in PNa (Sterns et al., 2010; Sood et al., 2013; MacMillan et al., 2015). There are different rates of 3% hypertonic saline that have been suggested. The US guidelines suggest a bolus of 100 mL over 10 min with three repeated doses as needed for severe symptoms and a rate of 0.5 to 2 mL/kg per hour for moderate symptoms (Verbalis et al., 2013). The European guidelines recommend a bolus of 150 mL over 20 min with 2–3 repeated doses for severe symptoms and a bolus of 150 mL over 20 min once for moderate symptoms (Spasovski et al., 2014). Some investigators have recommended the concomitant use of desmopressin 1 to 2 mcg intravenously or subcutaneously every 6 to 8 h in such patients at the outset to achieve a “clamp” on water diuresis and a syndrome of iatrogenic antidiuresis (Perianayagam et al., 2008). There are also reports of using 3% hypertonic saline in isolation or intravenous diuretics along with 3% hypertonic saline with variable success (Forssell et al., 1980; Woo et al., 2009).

### Reactive strategy

If water diuresis ensues during the treatment of hyponatremia and the initial rate of PNa improvement is fast enough that there are concerns that it may overshoot the recommended limit of PNa correction, then the reactive strategy of management can be employed. The patients who are at increased risk of rapid correction include those in whom the underlying reversible cause of hyponatremia is quickly corrected. These scenarios include cessation of drugs causing syndrome of inappropriate antidiuretic hormone (SIADH) or diuretics that interfere with urinary dilution, treatment of adrenal insufficiency with glucocorticoids, or true volume depletion with isotonic solution, and use of a vasopressin receptor antagonist to achieve water diuresis. (Table 1) This strategy involves either replacement of urinary‐free water losses with intravenous D5W (which is cumbersome) or preferably the administration of desmopressin 2 to 4 mcg intravenously or subcutaneously every 6 to 8 h to stop the water diuresis (Sterns et al., 2010; Sood et al., 2013; MacMillan et al., 2015; Sterns, 2018) (Table 2 and Fig. 2).

### Rescue strategy

When the correction of hyponatremia has overshot the recommended safe limit, the rescue strategy should be instituted swiftly. This involves the administration of desmopressin 2 to 4 mcg intravenously or subcutaneously every 6 h effectively stopping any water diuresis, and the infusion of D5W at the rate of 6 mL/kg lean body weight over 2 hours to re‐lower the PNa below the limit of correction.(Perianayagam et al., 2008; Sterns et al., 2010; Sood et al., 2013; Rafat et al., 2014; MacMillan et al., 2015) This rate of D5W is expected to lower the PNa by ≈1 mmol/L per hour in the absence of water diuresis under the effect of desmopressin, which may take some time to take effect (Table 2 and Fig. 2). This can be continued until the PNa level is re‐lowered to the desired therapeutic level. After lowering the PNa to the desired level, D5W is stopped but desmopressin should be continued as water diuresis may recur in its absence.

## Conclusion

The treatment of severe hyponatremia requires a methodical approach with close monitoring of PNa levels for appropriate rates of correction. Although there are disagreements on the optimal goals and limits of correction of plasma sodium, recommended limits of correction are as follows: a) an increase of < 10–12 mmol/L in the first 24 h and < 18 mmol/L in the first 48 h per US and European guidelines on hyponatremia management, or b) an increase in PNa ≤ 8 mmol/L in any 24 h‐period especially in the presence of other risk factors for ODS. We advocate for a goal of 4 to 6 mmol/L per day for patients with severe symptomatic hyponatremia. Patients with severe symptomatic hyponatremia that is of unknown duration should be treated as chronic, as inadvertent rapid correction may lead to the irreversible and potentially fatal complications of ODS. Hypoxic episodes, such as seizures, increase the risk of ODS in these patients. Prompt treatment is necessary for patients who are at high risk for brain herniation and death. Patients may be managed by following a proactive strategy (for patients with PNa < 120 and those known to be at high risk of overcorrection and ODS), reactive strategy (for patients who have an initial rapid trajectory of improvement worrisome for overshooting the therapeutic limit), or rescue therapy (for patients who have already overshot their therapeutic limit in an effort to minimize and prevent ODS by re‐lowering the PNa).

## Conflict of Interest

The authors have no conflict of interest to disclose.
